# Prognostic value of volume-based metabolic parameters of [^18^F]FDG PET/CT in anaplastic thyroid carcinoma

**DOI:** 10.1007/s00259-026-08100-0

**Published:** 2026-07-30

**Authors:** H. I. Coerts, O. P. Smit, J. F. Lin, P. van Houten, T. P. Links, A. H. Brouwers, C. C.D. van der Rijt, T. M. van Ginhoven, R. T. Netea-Maier, M. J.C. van Treijen, M. Medici, B. de Keizer, S. Kruijff, F. A. Verburg

**Affiliations:** 1 Academic Center for Thyroid Diseases, Department of Radiology and Nuclear Medicine, Erasmus Medical Centre Rotterdam, P.O. 2040, Doctor Molewaterplein 40, 3015 GD , CA 3000 Rotterdam, the Netherlands; 2 Department of Surgical Oncology, University of Groningen, University Medical Center Groningen, Hanzeplein 1, Groningen, 9713 GZ The Netherlands; 3 Department of Internal Medicine, Division of Endocrinology, Radboud University Nijmegen Medical Center, PO Box 9101, Nijmegen, 6500HB the Netherlands; 4 Division of Endocrinology, Department of Internal Medicine, University of Groningen, University Medical Center Groningen, Groningen, the Netherlands; 5 Department of Nuclear Medicine and Molecular Imaging, University of Groningen, University Medical Center Groningen, Groningen, the Netherlands; 6 Academic Center for Thyroid Diseases, Department of Medical Oncology, Erasmus MC Cancer Institute, Rotterdam, the Netherlands; 7 Academic Center for Thyroid Diseases, Department of Surgical Oncology and Gastrointestinal Surgery, Erasmus MC Cancer Institute, Rotterdam, 3015GD The Netherlands; 8 Department of Endocrine Oncology, University Medical Center Utrecht, Utrecht, 3584 CX the Netherlands; 9 Academic Center for Thyroid Diseases, Department of Internal Medicine, Erasmus Medical Center, Rotterdam, The Netherlands; 10 Department of Radiology and Nuclear Medicine, University Medical Center Utrecht, Utrecht, 3584 CX the Netherlands; 11 Department of Nuclear Medicine and Molecular Imaging, Medical Imaging Center, University Medical Center Groningen, Groningen, the Netherlands; 12 Department of Molecular Medicine and Surgery, Karolinska Institutet, Stockholm, Sweden

**Keywords:** Anaplastic Thyroid Carcinoma, FDG PET/CT, Metabolic tumor volume, Total lesion glycolysis, Prognosis

## Abstract

**Introduction:**

Anaplastic thyroid carcinoma (ATC) is a rare and aggressive malignancy with a median overall survival (OS) of approximately 2 months. The prognostic value of ^18^F-fluorodeoxyglucose positron emission tomography/computed tomography ([^18^F]FDG PET/CT) in ATC remains unclear. This study aims to evaluate the prognostic significance of [^18^F]FDG PET/CT parameters in ATC patients.

**Methods:**

This retrospective study analyzed 34 patients diagnosed with ATC between January 2010 and October 2024 who underwent initial staging with [^18^F]FDG PET/CT. Data collected included standardized uptake value (SUV), metabolic tumor volume (MTV), and total lesion glycolysis (TLG), along with patient and tumor characteristics. The relationships between these parameters and survival were assessed.

**Results:**

OS was 48 days (IQR: 27–134). In univariate analysis, MTV (HR = 1.44, *p* = 0.009) was significantly associated with poorer OS. Surgery was associated with a significantly decreased hazard of mortality (HR, 0.418; 95% CI, 0.188–0.929; *P* = 0.032), as was radiotherapy (HR, 0.423; 95% CI, 0.220–0.977; *P* = 0.026).

**Conclusion:**

This study identifies MTV as a significant prognostic markers in univariate analysis for patients with ATC, highlighting the utility of [^18^F]FDG PET/CT in predicting patient outcomes.

## Introduction

Anaplastic thyroid carcinoma (ATC) is a rare disease with low survival rates. The incidence rate is around 35 patients per year in the Netherlands [1]. The 5 year-survival is 8.4% and the median overall survival (OS) is reported to be 2.2 months in the Netherlands [1–3]. Survival rates have not improved significantly from 1986 to 2015, stressing the importance of research in this field [3]. When initially diagnosed, ATC has already metastasized to the lungs, bone, skin and brain in about 40% of cases [1, 2].

For stage IVA (cancer is found in the thyroid; may have spread to the lymph nodes), ATC treatment focuses on complete surgical resection when possible, often followed by external beam radiation therapy and chemotherapy to enhance local control; tracheostomy may be required if the airway is compromised [4]. In stage IVB, where the tumor extends beyond the thyroid or involves regional lymph nodes, surgery is pursued if feasible, followed by radiation and/or chemotherapy, again with airway management as needed. For stage IVC, which involves distant metastases, the approach shifts to palliation; surgery is typically limited to relieving symptoms, and systemic therapy becomes central. This may include chemotherapy, radiation, and targeted therapy, particularly with BRAF and MEK inhibitors like dabrafenib and trametinib in tumors harboring the BRAF V600E mutation. BRAF(V600E) and RAS mutations are the main drivers in ATC, which can be accompanied with TERT promotor, TP53, PIK3CA-PTEN-AKT-mTOR pathway, SWI-SNF complex, histone methyltranferases and MMR mutations [5]. Recent molecular testing has identified new mutation-targeted therapies [6]. Accurate staging is crucial for selection of the appropriate therapy. According to the 2021 American Thyroid Association (ATA) guidelines the ^18^F-fluorodeoxyglucose ([^18^F]FDG) positron emission tomography and computed tomography (PET/CT) has been recommended for initial staging of the disease (IVA, IVB or IVC) [4, 7]. As there is an increase in the glucose transporters 1 (GLUT1) gene in ATC, a higher [^18^F]FDG uptake is seen [8, 9]. An overexpression of GLUT1 is closely related to more aggressive behavior [10].

In a study of 40 patients with ATC, [18 F]FDG PET/CT was used to assess tumor glycolysis through parameters such as maximum standard uptake value (SUV_max_), metabolic tumor volume (MTV), and total lesion glycolysis (TLG) [11]. High values of SUV_max_, MTV, and TLG were each significantly associated with poor OS (*p* < 0.001, *p* = 0.002, and *p* < 0.001, respectively), indicating that increased glucose metabolism correlates with more aggressive disease. Patients with higher tumor SUV_max_ had significantly shorter survival, demonstrating that these PET-derived parameters serve as strong prognostic biomarkers in ATC.

Due to the low incidence rate of ATC, insufficient data is available on prognostic factors. The aim of this study is to determine if preoperative volume-based metabolic parameters on [^18^F]FDG PET/CT have prognostic value in patients with anaplastic thyroid cancer.

## Methods

### Study population

Patients diagnosed with ATC between January 2010 and October 2024 and who underwent [^18^F]FDG PET/CT for initial staging were selected for the study, as selected from an existing national database. The study was conducted across four academic hospitals in the Netherlands: Erasmus University Medical Center, Utrecht Medical Center, Radboud University Medical Center, and University Medical Center Groningen. Patients underwent [^18^F]FDG PET/CT imaging before any surgical intervention or radiotherapy. Included were all patients with histologically confirmed ATC who underwent [^18^F]FDG PET/CT imaging at any of the participating centers. Exclusion criteria included (1) poor image quality precluding accurate SUV measurements and (2) PET/CT scans limited to head and neck region only (without whole-body imaging). OS was defined as the time from the date of the initial [^18^F]FDG PET/CT scan to the date of death from any cause or last follow-up. The [^18^F]PET/CT date was used as the survival starting point rather than tissue diagnosis date to ensure temporal alignment between the prognostic parameters (MTV, TLG, SUV_max_) and survival outcomes, as these metabolic measurements represent tumor burden at the specific moment of PET/CT acquisition, not at the earlier diagnosis date. Patients alive at last follow-up were censored at that date.

### Imaging protocol and data acquisition

[^18^F]FDG PET/CT scans were obtained using different PET/CT systems at each participating hospital. A summary table of scanning protocols of each hospital can be found in Supplementary Table 1. The [^18^F]FDG PET/CT images were independently reviewed by three people, FV, HC and OS. Imaging analysis was performed using HERMES Medical Solutions software. The images were uploaded, and the post-treatment tumor finder tool was utilized to identify ATC lesions. The tumor in the thyroid bed was analyzed, lymph nodes and distant metastases. Standardized Uptake Values (SUV) were calculated using lean body mass (LBM) correction based on the James formula. Quantitative measurements included SUV_max_, metabolic tumor volume (MTV), and total lesion glycolysis (TLG). For determination of the SUV_mean_, a volume of interest (VOI) was delineated using a region growing algorithm with a fixed threshold at 50% of the maximum PET signal (SUV_max_). In cases of discrepancies between the reviewers, a consensus was reached through joint discussion. All measurements were obtained without knowledge of patient outcomes.

### Statistical analysis

Cox proportional hazards regression model was used to evaluate the association between imaging parameters and survival time in days, yielding hazard ratios (HRs) with 95% confidence intervals (CIs). Hazard ratios for MTV and TLG were expressed per 100 mL and per 1000 g increment, respectively, to reflect clinically meaningful differences in metabolic tumor burden across the observed distribution of values in our cohort [12]. Statistical significance was defined as a p-value < 0.05. All statistical analyses were performed using SPSS Statistics (IBM Corp.) and R software. Reporting was according to the STROBE guidelines [13].

## Results

### Baseline characteristics

A total of 36 patients met the inclusion criteria, of which 2 were excluded because only a head and neck scan was available. A total of 34 patients with ATC were included in the study. Baseline characteristics are shown in Table 1. The study cohort included 34 patients, of whom 44.1% (*n* = 15/34) were female. The mean age was 71.7 years (± 11.3). Median survival was 48 days (IQR: 27–134) after [^18^F]FDG PET scan. The 1 year overall survival (OS) was 8.8% (*n* = 3/34). In terms of treatment, 35.3% (*n* = 12/34) underwent surgery, 38.2% (*n* = 13/34) received radiotherapy, and 8.8% (*n* = 3/34) were treated with tyrosine kinase inhibitors (TKIs). Regarding disease localization, 14.7% (*n* = 5/34) had only localized disease, 23.5% (*n* = 8/34) had lymph node metastases, and 61.8% (*n* = 21/34) had distant metastases. Based on TNM classification, 14.7% (*n* = 5/34) were N0M0, 23.5% (*n* = 8/34) N1M0, 14.7% (*n* = 5/34) N0M1, and 47.1% (*n* = 16/34) N1M1. Most patients were diagnosed with stage IVc disease (79.4%, *n* = 27/34), while 14.7% (*n* = 5/34) were stage IVb and 5.9% (*n* = 2/34) were stage IVa.

**Table 1:** Baseline Characteristics of Included Patients

Variable	(*n* = 34)
Sex (female)	15/34 (44.1%)
Age (mean ± SD)	71.66 ± 11.34
Survival time (median, IQR; in days)	48 [27.25-134.25]
**Localisation of Disease**	
Only Localized Disease	5/34 (14.7%)
Lymph Node Metastasis	8/34 (23.5%)
Distant Metastasis	21/34 (61.8%)
**TNM Classification**	
N0, M0	5/34 (14.7%)
N1, M0	8/34 (23.5%)
N0, M1	5/34 (14.7%)
N1, M1	16/34 (47.1%)
**Therapy**	
No treatment	13 (38.2%)
Radiotherapy only	7 (20.6%)
Surgery only	6 (17.6%)
Surgery + Radiotherapy	5 (14.7%)
Surgery + Radiotherapy + TKI	1 (2.9%)
Surgery + TKI	1 (2.9%)
TKI only	1 (2.9%)

Categorical variables are presented as n/N (%). Age is presented as mean ± standard deviation. Survival time is presented as median [interquartile range] in days. Abbreviations: IQR, interquartile range; M, distant metastasis; N, lymph node metastasis; SD, standard deviation; TKI, tyrosine kinase inhibitor; TNM, tumour-node-metastasis

**Fig. 1:**
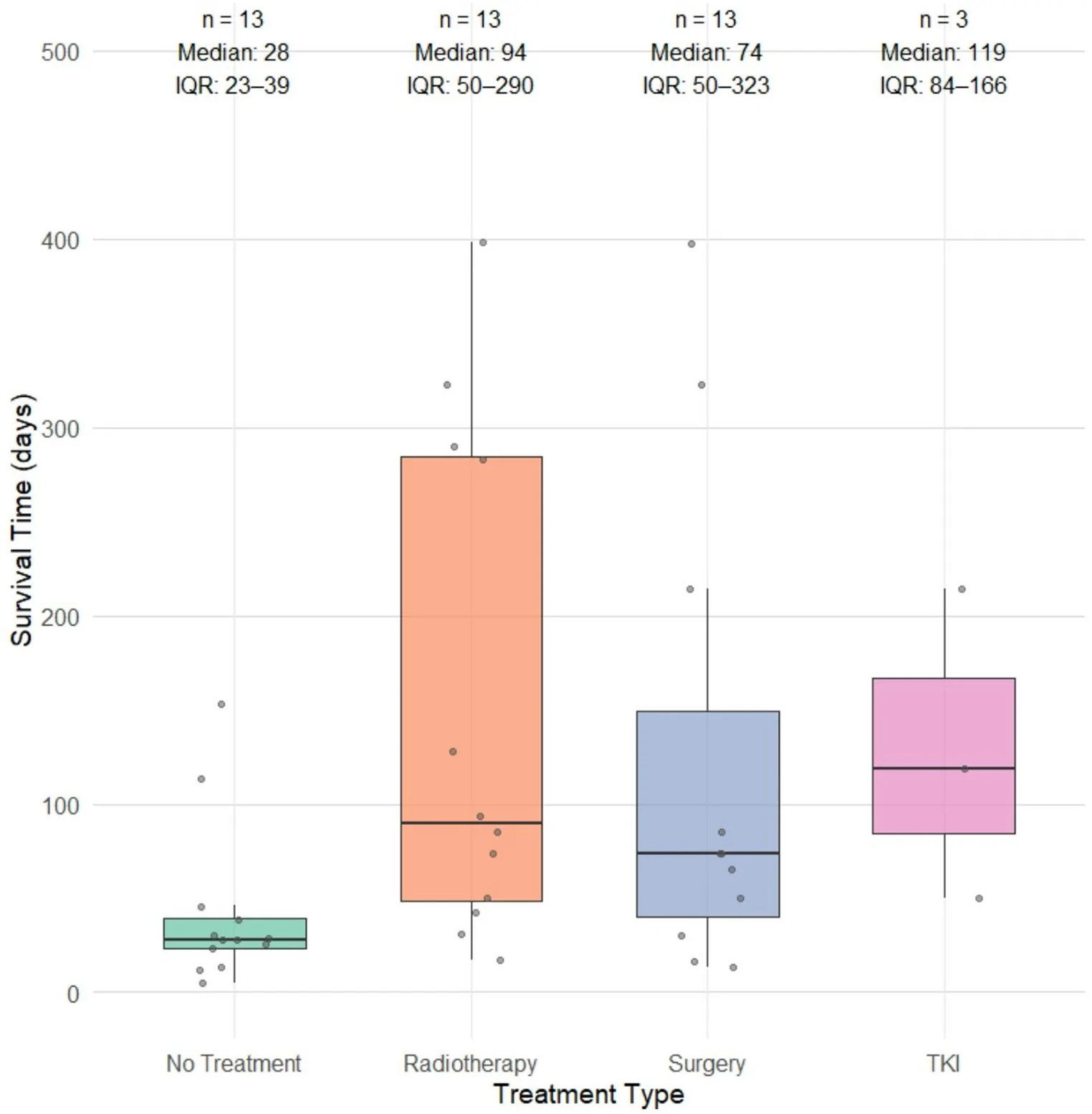
Boxplot of survival time by treatment modality. *TKI = Tyrosine Kinase Inhibitor*

**Fig. 2:**
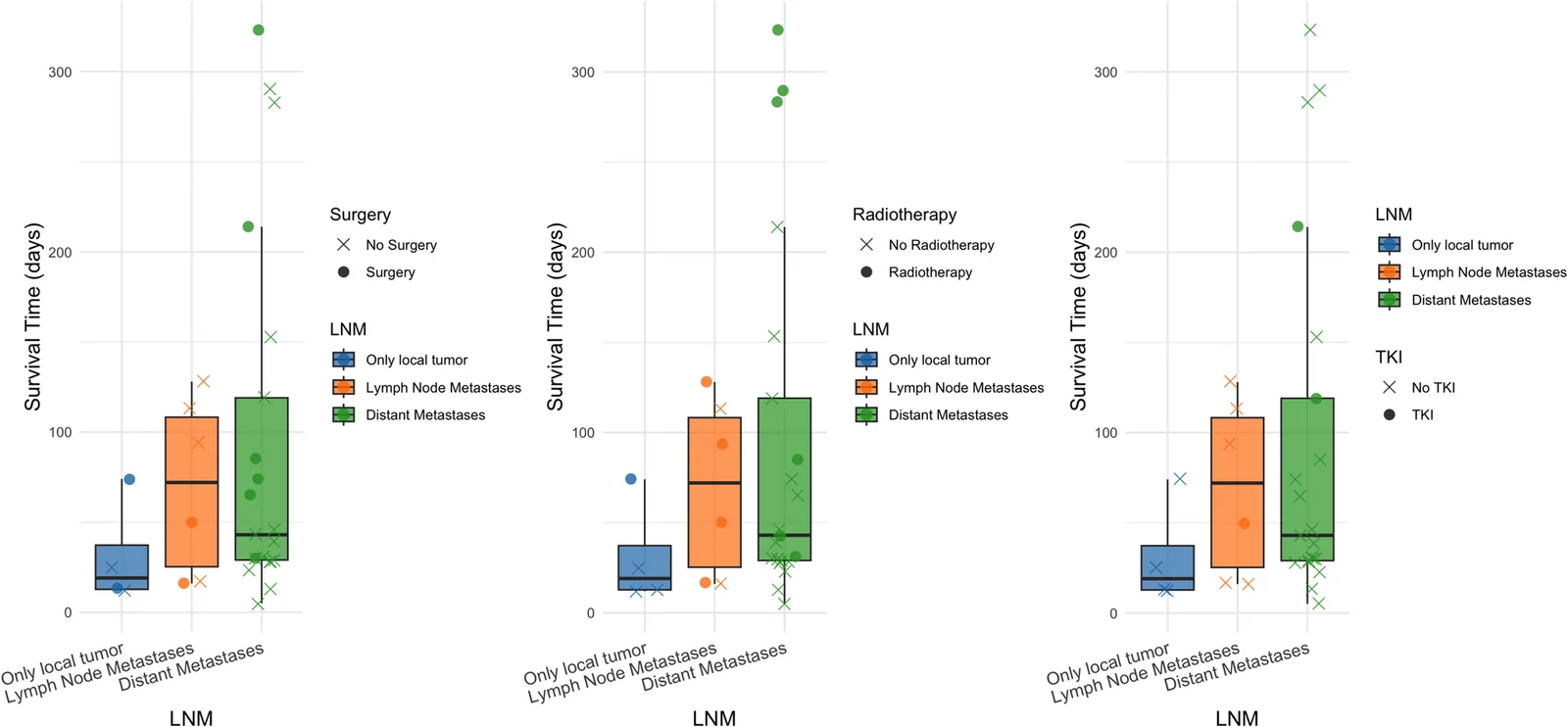
Boxplot of survival time by disease status and type of treatment. *LNM = Lymph Node Metastasis; TKI = Tyrosine Kinase Inhibitor*

Median survival times varied across treatment modalities (Fig. 1). Patients receiving no treatment (*n* = 13) had the shortest median survival time of 28 days (interquartile range [IQR]: 23–39). Median survival was longest in the TKI group (*n* = 3) at 119 days (IQR: 84–166). Radiotherapy (*n* = 13) was associated with a median survival of 94 days (IQR: 50–290), while surgery (*n* = 13) had a median survival of 74 days (IQR: 50–323). Individual survival times showed considerable variability within each group, as indicated by the range and presence of outliers. Survival times by presence of lymph node and distant metastases and treatment modality are depicted in the grouped boxplots (Fig. 2). Across all three types of treatment, patients with only local tumors had the shortest survival times, while those with distant metastases exhibited the longest median survival times, though considerable variability and overlapping ranges were observed within each group.

**Fig. 3:**
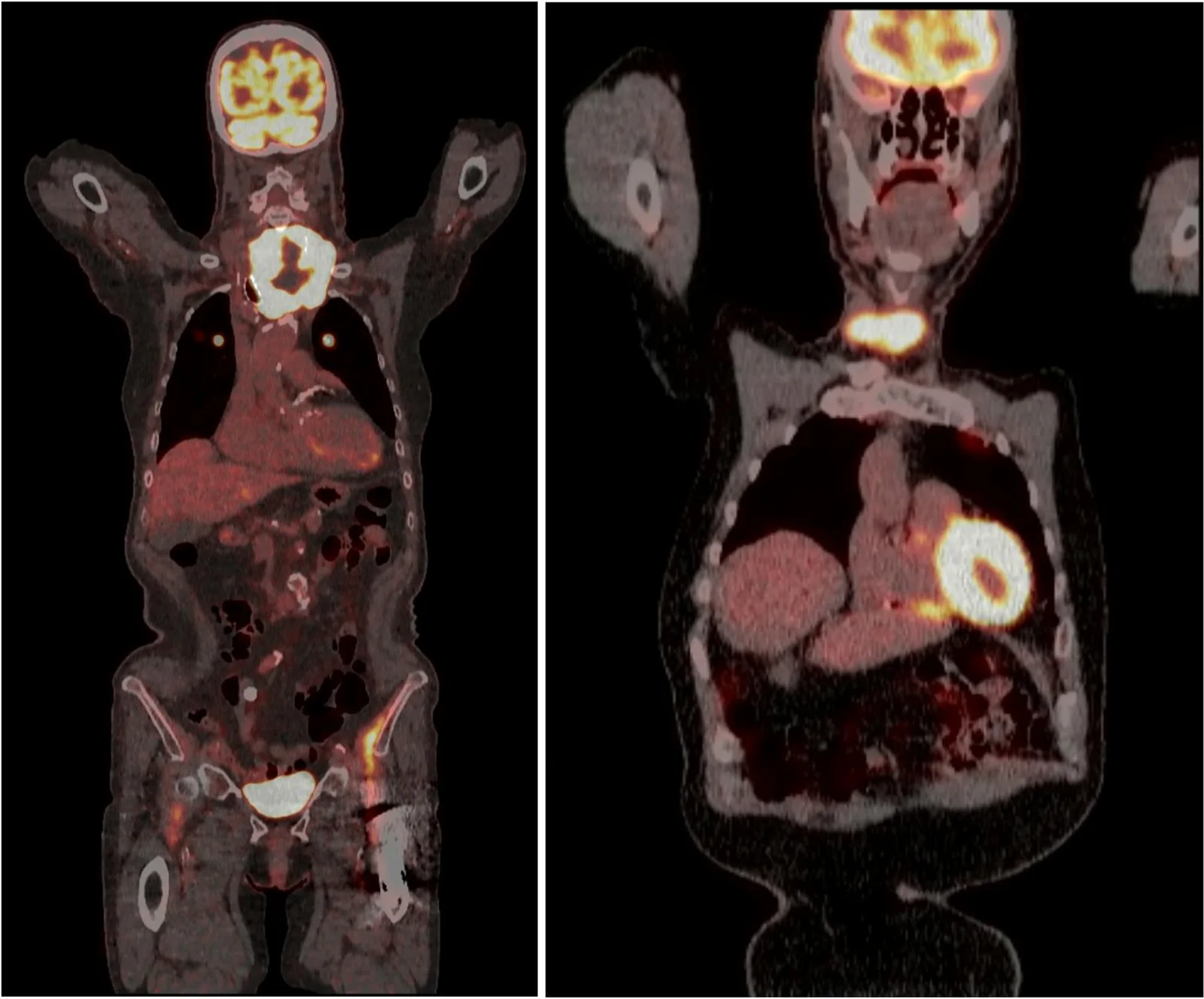
Representative [¹⁸F]FDG PET/CT images of two ATC patients with contrasting metabolic tumor burden. Left: patient with high MTV (373 mL) and survival of 13 days. Right: patient with low MTV (57 mL) and survival of 153 days

Metabolic parameters were assessed and compared across patient groups based on survival time and treatment modality (Tables 2 and 3). Representative [¹⁸F]FDG PET/CT images illustrating the spectrum of metabolic tumor burden across patients are shown in Fig. 3. When stratified by survival time, patients with a survival of less than one month (*n* = 13) demonstrated the highest median MTV [219 mL(IQR: 171–364), range 14–400], while those surviving four or more months (*n* = 9) had the lowest MTV [51 mL (33–148), range 13–399], though the difference did not reach statistical significance (*p* = 0.118). Evaluation based on treatment modality showed that patients who received no treatment had the highest median MTV [234 mL (162–374), range 54–508], while those treated with TKIs had the lowest [96]. Similarly, median SUV_max_ values tended to be higher in the no treatment and radiotherapy groups compared to patients treated with surgery or TKIs. TLG followed a comparable pattern, with the *no treatment* group demonstrating the highest values.

**Table 2:** Metabolic Parameters per Group based on Survival Time

	All patients (*n* = 30)*	Survival time < 1 month (*n* = 12)	Survival time 1–4 months (*n* = 10)	Survival time 4 + months (*n* = 8)	*p*-value
Thyroid gland					
**MTV**	156 [56–328] (13, 400)	219 [171–364] (14, 400)	121 [71–248] (51, 388)	51 [33–148] (13, 399)	*0.118*
**SUV**_**max**_	26 [19–35] (4, 75)	21 [18–29] (11, 54)	32 [21–44] (13, 75)	25 [23–34] (4, 37)	*0.326*
**TLG**	1048 [499–2782] (69, 8105)	1312 [894–3220] (82, 4340)	982 [795–1646] (250, 8105)	383 [276–2113] (69, 4886)	*0.467*
**Total body**					
**MTV**	188 [89–346] (13, 508)	280 [189–364] (54, 508)	146 [101–252] (51, 472)	65 [25–218] (13, 399)	*0.063*
**SUV**_**max**_	28 [18–36] (4, 74)	23 [16–32] (11, 54)	35 [22–46] (13, 74)	27 [23–32] (4, 37)	*0.251*
**TLG**	1158 [796–2799] (69, 8685)	1510 [1052–3227] (255, 4641)	1098 [796–1765] (249, 8685)	602 [240–2109] (69, 5168)	*0.333*

Data are presented as median [interquartile range] (minimum, maximum). P-values were calculated using the Kruskal-Wallis test. Abbreviations: MTV, metabolic tumour volume; SUV, standardised uptake value; TLG, total lesion glycolysis. * Four patients without whole-body PET/CT data were excluded from this analysis

**Table 3:** Metabolic Parameters for Total Body per Group based on Treatment Modality*

MTV	No Treatment	Radiotherapy	Surgery	TKI
	234 [162–374] (54, 508)	96 [48–200] (23, 399)	142 [76–286] (13, 396)	96 [96–96] (96, 96)
**SUV**_**max**_	27 [18–37] (11, 74)	30 [19–36] (4, 54)	30 [23–34] (4, 39)	34 [34–34] (34, 34)
**TLG**	1434 [966–4152] (255, 8685)	817 [310–1446] (69, 4886)	1098 [696–1616] (69, 5168)	1061 [1061–1061] (1061, 1061)

* Some patients received both surgery and radiotherapy. Due to this overlap between treatment groups, standard statistical tests assuming independent groups were not applicable, and thus no p-value is reported

Data are presented as median [interquartile range] (minimum, maximum). P-values were calculated using the Kruskal-Wallis test. Abbreviations: MTV, metabolic tumour volume; SUV, standardised uptake value; TLG, total lesion glycolysis

### Univariate analysis

The Cox proportional hazards regression model results are shown in Table 4 (all patients) and 5 (patients without intervention). In univariate analysis, MTV (per 100 mL) was significantly associated with increased hazard of mortality (HR, 1.44; 95% CI, 1.10–1.89; *p* = 0.009). Surgery was associated with a significantly decreased hazard of mortality (HR, 0.42; 95% CI, 0.19–0.93; *P* = 0.033), as was radiotherapy (HR, 0.42; 95% CI, 0.20–0.90; *p* = 0.025). Other metabolic parameters, such as SUV_max_ and TLG, did not demonstrate significant associations with survival.

**Table 4:** Cox Proportional Hazards Regression Model on Survival Time

	Test for Survival Time	Hazard Ratio	*P*	95% CI
Univariate analysis				
MTV (per 100 mL) thyroid	Continuous	1.44	0.009	1.10–1.89
SUV_max_ thyroid	Continuous	1.00	0.751	0.98–1.03
TLG (per 1000) thyroid	Continuous	1.12	0.191	0.95–1.32
MTV (per 100 mL) whole body	Continuous	1.44	0.005	1.11–1.86
SUV_max_ whole body	Continuous	1.01	0.685	0.98–1.03
TLG (per 1000) whole body	Continuous	1.12	0.159	0.96–1.32
LN metastasis	Present vs. absent	1.14	0.787	0.43–3.03
Distant metastasis	Present vs. absent	1.91	0.126	0.83–4.38
Surgery		0.42	0.033	0.19–0.93
Radiotherapy		0.42	0.025	0.20–0.90
TKI		1.38	0.756	0.18–10.38

*Multivariate analysis was not performed due to insufficient events; Hazard ratios for MTV are reported per 100 mL increase; hazard ratios for TLG per 1000-unit increase. Hazard ratios for SUVmax and age are reported per unit increase. P-values < 0.05 are considered statistically significant. Abbreviations: CI, confidence interval; HR, hazard ratio; LN, lymph node; M, distant metastasis; MTV, metabolic tumour volume; N, lymph node metastasis; SUV, standardised uptake value; TKI, tyrosine kinase inhibitor; TLG, total lesion glycolysis; TNM, tumour-node-metastasis

### Treatment patterns and survival outcomes in patients stratified by disease extent

Of all patients who received [^18^F]FDG PET/CT, 58.8% (*n* = 20/34) of patients received surgery or radiotherapy. Among patients with only localized disease (*n* = 5), 2 patients received no intervention, 2 patients underwent surgery plus radiotherapy, and 1 patient had surgery alone. For patients with lymph node metastases, without distant metastasis (*n* = 8), radiotherapy was the most common treatment (*n* = 3), followed by surgery (*n* = 2) and surgery plus radiotherapy (*n* = 2), with only 1 patient receiving no intervention. Among patients with distant metastases (*n* = 21), the majority (52.4%) received no intervention, while 19.0% underwent surgery, 19.0% received radiotherapy, and 9.5% had a combination of surgery and radiotherapy. In the group with distant metastases, the patients who received intervention had a median overall survival of 80 days (IQR: 40–285 days). The patients who did not receive intervention had a median overall survival of 29 days (IQR: 23–46 days). Three patients received TKIs, one patient with lymph node metastases and 2 with distant metastases. The survival time in these patients was 50, 119 and 214 days.

### Patients with > 1 year survival

A total of three patients had a survival > 1 year. Their clinical characteristics are shown in Table 5. All three patients had MTV < 60 mL and TLG < 605 g.

**Table 5:** Patient Characteristics of Patients Survival > 1 Year

#	Survival time in days	Treatment	TNM	Vital status	MTV	SUV_max_	TLG	Age at diagnosis	Sex
1	2934	Surgery + RTx	T4aN1bM0	Alive	44	25	335	47	Male
2	1705	Surgery	T4aN1bM0	Alive	13	25	100	77	Male
3	398	Surgery + RTx	T4aN0M0	Alive	23	4	69	62	Male

*RTx* Radiotherapy, *TNM* , Tumor, node, metastasis, *MTV* Metabolic tumor volume, *SUV*_*max*_ Maximum standard uptake value, *TLG* Total lesion glycolysis, *RTx* Radiotherapy

## Discussion

In this multicenter retrospective cohort study of patients with ATC we identified MTV as a significant prognostic indicator of survival.

PET/CT is a valuable tool in diagnosis and treatment planning for patients with ATC, as it is the most sensitive imaging technique for detecting extent of disease [14]. The extent of disease may play a role in choosing between treatment modalities, suggesting a two-step approach in treatment selection. Further exploration of volume-based metabolic parameters on [^18^F]FDG PET/CT could enhance the ability to assess the aggressiveness of disease, providing even more precise information for guiding therapy and improving patient outcomes.

Our analysis revealed that higher MTV values are associated with shorter survival outcomes, which is consistent with previous studies suggesting that a higher metabolic burden correlates with increased tumor aggressiveness in ATC [11, 14]. The utility of PET/CT-derived metabolic parameters, such as MTV and TLG, has been previously examined in various cancers, though data on ATC has been limited. This study contributes valuable evidence supporting the prognostic relevance of MTV and TLG in this aggressive malignancy. Importantly, while SUV_max_ has been a commonly used prognostic marker in other cancers, it did not emerge as a significant predictor in our cohort. SUV_max_ reflects the highest FDG uptake within a single voxel, making it highly susceptible to technical and biological variability, such as image noise, scanner resolution, and inflammatory activity. As a result, it may not accurately represent the overall metabolic activity of the tumor, particularly in highly heterogeneous malignancies like ATC, which frequently contain necrotic or poorly perfused areas. In contrast, volumetric parameters such as MTV provides a more comprehensive assessment of the entire tumor burden and its metabolic activity. These parameters may therefore be more robust and informative prognostic markers in ATC, where tumor heterogeneity plays a significant role in clinical outcomes [11, 14].

Three previous studies, involving a total of 68 patients, have examined the relationship between [^18^F]FDG uptake and survival in ATC [7, 9, 10]. The study by Bogsrud et al. (*n* = 16) did not find any significant correlation between survival and SUV_max_ in primary tumors [9]. No hazard ratios or detailed statistical analyses were provided. In contrast, Poisson et al. (*n* = 20) identified both the volume of [^18^F]FDG uptake and intensity of [^18^F]FDG uptake (SUV_max_ ≥18, as significant prognostic factors [14]. Our study similarly found MTV to be of prognostic value. However, we disagree with their conclusion regarding SUV_max_, as we did not find it to be prognostic. In their bivariate analysis, only functional volume remained significant (*p* = 0.004), while SUV_max_ lost its prognostic power. No hazard ratios were reported by Poisson et al., and TLG was not assessed in their study. Notably, in our cohort, two out of three patients who survived longer than one year had a SUV_max_ >18, highlighting the variability in the prognostic utility of SUV_max_.

The most recent and largest study, conducted by Kim et al. (*n* = 40), found that high SUV_max_, MTV, and TLG were significantly associated with poor prognosis [11] Kim et al. observed significant differences in overall survival (OS) between patients with high and low SUV_max_ (*p* < 0.001), a finding that differs from ours. These differences may be attributed to the small sample size in both studies. Regarding treatment modalities, Kim et al. reported hazard ratios of 0.459 for surgery and 0.891 for radiotherapy, while our analysis found hazard ratios of 0.42 for surgery and 0.42 for radiotherapy. Interestingly, we observed a stronger effect of radiotherapy, suggesting that radiotherapy might play a more crucial role in our specific cohort. However, radiotherapy would be less likely selected for patients experiencing severe tracheal lumen narrowing due to tumor compression. As a result, it is often patients with lower disease burden who receive these treatments. This introduces a significant selection bias, which is an inherent limitation in retrospective studies of this kind.

Regarding clinical characteristics, a recent French multicenter study involving 360 patients identified several factors independently associated with improved overall survival in ATC, including Eastern Cooperative Oncology Group (ECOG) status, disease stage (IVa, IVb, or IVc), multimodality treatment, synchronously transformed ATC, and lower neutrophil-to-lymphocyte ratio (NLR) [15]. However, disease stage did not emerge as a significant factor in our cohort, likely because most of our patients (27 out of 34) were at stage IVc. In our dataset, patients with distant metastases demonstrated longer median survival compared to those with only local disease, despite higher MTV and TLG being associated with worse outcomes in univariate analysis. This may reflect the predominance of locoregional disease progression as the primary mode of death in our cohort, rather than distant metastatic burden. Other hypotheses are that patients in our cohort who received aggressive multimodal treatment (surgery and/or radiotherapy ± TKI) had better baseline performance status, enabling them to tolerate intensive therapy and achieve longer survival even with distant metastases. Conversely, patients with locally advanced disease but poor performance status, due to comorbidities or rapid respiratory compromise. received palliative care only, resulting in very short survival regardless of their metabolic parameters. This treatment selection bias makes it challenging to disentangle the independent prognostic value of PET parameters from treatment effects. Ideally, prognostic biomarkers should be validated in homogeneously treated cohorts or using propensity score matching, approaches not feasible with our limited sample size.

### Limitations and future directions

This study has several important limitations that warrant consideration. First, the relatively small sample size (*n* = 34) represents a significant constraint. Post-hoc power considerations suggest that with 34 patients and the observed effect sizes, our study may have been underpowered to detect modest prognostic effects as statistically significant. Given the borderline p-values, type II error (false negative findings) cannot be excluded, and the true prognostic value of MTV, SUV_max_ and TLG may have been underestimated due to insufficient statistical power. However, ATC is exceedingly rare, representing less than 2% of all thyroid cancers, and our cohort represents one of the larger single-center series with baseline [18 F]FDG PET/CT data reported to date.

Second, treatment selection bias represents a major confounding factor in our analysis. Patients who received aggressive multimodal therapy (surgery and/or radiotherapy) likely had better baseline performance status and more favorable disease characteristics, independent of their metabolic parameters. This selection bias makes it challenging to disentangle the independent prognostic value of PET metrics from treatment effects. Our subgroup analysis of untreated patients (*n* = 13), while suggestive, was severely underpowered for definitive conclusions. Ideally, prognostic biomarkers should be validated in homogeneously treated cohorts or using propensity score matching to adjust for treatment allocation bias, approaches that are not feasible with our limited sample size.

Third, our study lacks systematic tumor size measurements, precluding direct comparison between MTV and conventional anatomical size assessments. While MTV represents volumetric metabolically active tumor burden and likely correlates with anatomical size, we cannot empirically demonstrate the added prognostic value of MTV over simple maximal tumor diameter measurements in our cohort.We present our findings as hypothesis-generating, providing preliminary evidence that warrants validation in larger, prospectively designed multicenter studies with sufficient statistical power to perform adjusted survival analyses.

## Conclusion

Our study highlights the importance of MTV as possible prognostic marker for ATC, underscoring the potential of [^18^F]FDG PET/CT in prediction of biological behavior and prognosis. Treatment modalities, particularly surgery and radiotherapy, showed independent benefits for patient survival. Given the differences observed between our findings and prior studies, larger and more diverse patient cohorts are warranted to further validate these prognostic factors and refine the management strategies for ATC.

## Data Availability

The datasets generated and/or analyzed during the current study are not publicly available due to patient privacy regulations but are available from the corresponding author on reasonable request and with appropriate ethical approval.
